# Undiagnosed hypertension and associated factors among long-distance bus drivers in Addis Ababa terminals, Ethiopia, 2022: A cross-sectional study

**DOI:** 10.1371/journal.pone.0292890

**Published:** 2024-02-15

**Authors:** Abebaw Bires Adal, Rahel Nega Kassa, Mekdes Hailegebreal Habte, Melkamu Getaneh Jebesa, Sewunet Ademe, Chalachew Teshome Tiruneh, Atsedemariam Andualem, Zewdu Bishaw Aynalem, Bekalu Bewket

**Affiliations:** 1 College of Medicine and Health Science, Department of Nursing, Injibara University, Injibara, Ethiopia; 2 Department of Nursing, St. Paul’s Millennium Medical College, Addis Ababa, Ethiopia; 3 Department of Care and Treatment, AIDS Health Care Foundation, Addis Ababa, Ethiopia; Rift Valley University, ETHIOPIA

## Abstract

**Introduction:**

Hypertension is a major public health problem that is often unrecognized, and its detection and control should be prioritized. The level of undiagnosed hypertension and its associated factors among long-distance bus drivers in Ethiopia is unknown.

**Objective:**

This study aimed to assess the magnitude of undiagnosed hypertension and its associated factors among long-distance bus drivers in Addis Ababa bus terminals.

**Methods:**

A facility-based cross-sectional study was conducted on 391 long-distance bus drivers from December 15, 2021, to January 15 2022 at five cross-country bus terminals in Addis Ababa. A standardized and structured questionnaire was adapted based on the WHO stepwise approach to a non-communicable disease study and translated into Amharic. Data were coded, cleaned, and entered using Epi-data version 4.6 and exported to SPSS version 26. Logistic regression analysis was performed. Variables with a P-value < 0.25 in the bivariable analysis were selected for multivariable logistic regression analysis. Independent variables with a P-value < 0.05 were considered statistically significant. The magnitude of association between independent and dependent variables was measured by odds ratio with a 95% confidence interval.

**Results:**

In this study, 391 study participants were involved with a response rate of 97.1%. The prevalence of undiagnosed hypertension was 22.5% (CI: 18.7%, 26.6%). Poor level of knowledge (AOR: 2.00, CI: 1.08, 3.70), long duration of driving per day (AOR: 2.50, 95% CI: 1.37–4.56), habit of chewing of chat (AOR: 2.61, 95% CI: 1.44, 4.73), regular alcohol consumption (AOR = 3.46; 95% CI: 1.70, 7.05), overweight (AOR:3.14, 95%CI: 1.54,6.42) obesity (AOR: 3.21, 95% CI 1.35, 7.61) and regular physical exercise (AOR: 0.16, 95% CI: 0.09, 0.29) were statistically significantly associated with undiagnosed hypertension.

**Conclusion:**

This study revealed that the prevalence of undiagnosed hypertension among long-distance bus drivers was 22.5%, which was associated with modifiable behavioral factors, lack of regular physical exercise, lack of adequate awareness and high body mass index.

**Recommendation:**

Stakeholders must implement the necessary preventive measures. These include increasing the level of awareness of hypertension among long-distance drivers and developing prevention of hypertension strategies and policies focusing on lifestyle and behavioral modifications.

## Introduction

High blood pressure, also known as hypertension (HTN), is a condition in which blood pressure increases on vessel walls [1]. It is similarly defined as systolic blood pressure (SBP) ≥ 140 mm Hg and/or diastolic blood pressure (DBP) ≥ 90 mmHg [2].

Hypertension remains a major public health problem and a leading contributor to cardiovascular death and disability [3]. One major cause of HTN’s high morbidity and mortality is its asymptomatic nature and suboptimal health [4]. Therefore, HTN is usually undiagnosed and untreated. It then causes chest pain, heart failure, arrhythmia, blurred vision, kidney failure, and stroke, which can lead to sudden premature death [5].

Globally, 41% of women and 51% of men, and approximately 580 million people with high blood pressure do not know they have elevated BP. Most HTNs are diagnosed during medical visits due to other health complaints [6].

Long-distance drivers are predisposed to high cardiovascular risk because of the sedentary nature of their work, illiteracy, and high-risk behaviors. Although their well-being has a great impact on the safety of passengers and road users, drivers place little emphasis on their self-sustained health care and neglect their health [7]. Different studies have also shown that the risk and prevalence of cardiovascular disease are higher among long-distance bus drivers than among other populations [7, 8].

Globally, approximately one billion people are affected by HTN, and this number is predicted to increase to 1.5 billion by 2025 [9]. It is estimated that it could result in 54% of all strokes, 47% of all ischemic heart disease, and 7.6 million premature deaths. Apart from its high prevalence, most individuals do not know or control their BP status [10]. According to the World Health Organization (WHO), there is a significant gap in the diagnosis of HTN and about 46% of adults with HTN are unaware that they have the condition [2].

The prevalence of HTN and remaining undiagnosed is higher in low-income and middle-income countries than in developed countries [11]. In 2015, 8.5 million deaths were associated with HTN, of which low-and middle-income countries contributed 88% [12]. Undiagnosed HTN contributes largely to the great burden of HTN in sub-Saharan Africa mainly because of poor health awareness, lack of access to services, and low socioeconomic status [13].

The 2021 report by WHO indicated that the prevalence of HTN in Ethiopia is 25% [6]. Different studies in Ethiopia also showed a higher prevalence of HTN in the general population and its estimated prevalence of 15.6% [14, 15]. It is also shown that a small percentage of people were aware of their BP status and the estimated prevalence of undiagnosed HTN in Ethiopia is 12.3% [16]. A recent study showed that HTN caused the highest medical admissions and 13.05% of all medical mortalities from its complications. There are 17.5% did not know they were hypertensive [17]. This indicates that more work is needed to identify the disease at an early stage.

It has been shown internationally and in Africa that HTN varies according to occupation, and drivers are among professionals for whom it is more prevalent [11, 18]. Long-distance bus drivers are a risk and target group for cardiovascular mortality and morbidity due to HTN. Additionally, it is shown that they are 3 times more likely to develop CVD than other drivers [8, 19, 20]. A systematic review and meta-analysis of 26 studies also showed that more than one-third of long-distance drivers had HTN [21].

This is mainly due to an increase in the prevalence of undiagnosed HTN [22–24]. This places them in a cluster of HTN complications and risk factors for CVDs such as silent myocardial infarction and stroke in the general population [8, 25, 26]. These complications are becoming a concern while driving and cause road traffic accidents [27, 28].

The identified causes of bus drivers’ vulnerability to HTN are; poor job satisfaction, long duration of working hours, insufficient breaks in between shifts, low levels of exercise, emotionally aggressive behavior towards busy traffic roads, and a blaring working environment [8, 28]. In addition, unawareness of their HTN risk behaviors such as alcohol consumption, smoking, regular high-calorie diets, and eating under stressful conditions are factors [25]. Because they lack awareness of the asymptomatic nature of HTN, their risky behavior, and their low socioeconomic status, they are becoming highly likely to live with HTN without diagnosis [22–24].

The World Health Organization states that automated devices can be used for self-measurement, but risk assessment and associated factors by health professionals remain the only appropriate approach to diagnose HTN [11]. In addition, risk reduction strategies such as prevention, early detection by targeting individuals at risk and control programs of HTN have been advocated to reduce the impact of HTN on life and economy [29].

From a few studies conducted among different communities in Ethiopia, identifying risk factors, reducing risky behavior by delivering health education and carrying out timely BP check-ups are recommended strategies to reduce the burden of undiagnosed HTN. [16, 30, 31]. In 2020, the Federal Ministry of Health Ethiopia (FMoH) tried to set a new program for the improvement of the control and prevention of HTN at primary health facilities in which screening and diagnosis are the components [32]. However, to my knowledge, the implementation of these measures is poor for bus drivers in Ethiopia. In other countries: annual health checkups, early screening, periodic HTN campaigns, onsite clinics, and health education are the recommended means to detect HTN among bus drivers [22, 33]. In addition to this, as we have experienced at health facilities, many stroke patients worked as long-distance drivers.

In Ethiopia, there is no adequate effort by stakeholders to provide these strategies, including the Ministry of Transport (MoT). In addition, adequate research has not been conducted to explore the PIS level for HTN in Ethiopia. Therefore, this study aimed to determine the magnitude of undiagnosed HTN and its associated factors among long-distance bus drivers in Addis Ababa terminals in Ethiopia.

## Methods and materials

### Study area and period

This study was conducted at cross-country bus stations (terminals) in Addis Ababa, Ethiopia. Addis Ababa is the capital city of Ethiopia and the African Union. It is the largest city in Ethiopia, with a population of 5,006,000 according to the 2021 Addis Ababa, Ethiopia Metro Area Population [34]. There are 5 cross-country bus terminals in Addis Ababa and 752 long-distance bus drivers whose initial point is in Addis Ababa. They serve as initial and destination points for many passengers across the country. According to the information gathered from terminal logistics, approximately 1000 to 1600 cross-country passengers are served each day by these terminals which are Autobis tera, Asko, Lamberet, Kality and Ayer Tena Bus terminals. In addition, there are 318 long-distance cross-country buses locally known as Autobus and the carrying capacity of each bus ranges from 60 to 70 passengers. This study was conducted from December 15 2021 to January 15, 2022.

### Study design and population

A facility-based cross-sectional study was performed on professional bus drivers who travel within a radius greater than 200 km from Addis Ababa. The source population was long-distance bus drivers at an initial point in Addis Ababa, Ethiopia. The study population was all sampled long-distance passenger bus drivers who were registered on a file of private road transport unions at selected terminals and travelled at a distance of greater than 200 km per day from Addis Ababa [33, 35], The study units were individual long-distance bus drivers.

#### Inclusion and exclusion criteria

Long-distance bus drivers who had a professional driving license and were constantly working full-time at each station were included. However, long-distance bus drivers who were severely ill and those with a working experience of less than 6 months were excluded from this study.

#### Participants’ behavior characteristics

*Physical activity*. A Participant was considered physically active if he could perform aerobic exercise (such as walking, biking, dancing and racing) or anaerobic activity (body building) for at least 150 minutes a week.

*Smoking*. A participant was an active smoker if he used tobacco regularly at a minimum of once per week in the previous 12 months.

*Alcohol*. A participant was an alcohol consumer if he drank any alcoholic beverages regularly per week in a minimum of one portion of alcohol such as 1 glass of wine, 1 bottle of beer, 50 g of ouzo or the like.

### Sample size determination

The sample size for the first specific objective was calculated by using a single population formula by considering: the proportion of the magnitude of undiagnosed hypertension (38.7%) from a similar study conducted in Ghana in 2020 [33]. The margin of error (d = 0.05), level of significance (α = 0.05), Z α /2 at 95% CI = 1.96, and 10% contingency rate. In addition, the sample sizes for the second specific objective were calculated by the double population proportion formula using epi data; for different associated variables like age, knowledge, smoking, experience and consumption of alcohol by using open epi-data and by considering: the prevalence of the problem among exposed and unexposed to undiagnosed hypertension and odds ratio from pieces of literature, exposed to an unexposed ratio of 1 as it is cross-sectional study and also power is 80%. (Table 1) and compared with the first specific objective sample size.

**Table 1:** Sample size for different independent variables of long-distance bus drivers, Addis Ababa bus terminals, Ethiopia, 2021 (n = 391).

S.no	Variables	% of undiagnosed HTN in exposed	% of undiagnosed HTN in unexposed	Exposed to unexposed ratio	OR	Non-response rate	Sample size Using 95% CI	Reference
1	Age 50–69	60.8%	39.2%	1	5.09	10%	184	[33]
2	Experience greater than 10	71.4%	7.3%	1	3	10%	290	[33]
3	Knowledge	18.7%	6%	1	6.2	10%	240	[46]
4	Alcohol	76%	24%	1	3.19	10%	34	[33]
5	Smoking	20.1%	79.9%	1	7.09	10%	26	[33]
6	FH of HTN	23%	76.5%	1	2.7	10%	32	[30]

### The sample size for the first objective


n=(Zα2)2P(1−P)d2

Where *n*_0_ = sample size, Zα_/2_ = 1.96, p = proportion of the magnitude of undiagnosed hypertension from the last literature which is 38.7%, d = margin of error.


$$n=\frac{{\left(1.96\right)}^{2}0.387(0.613)}{0.05^{2}}=365$$

By adding a 10% non-response rate, the final sample size was 402.

### Sampling techniques and procedures

**Fig 1:**
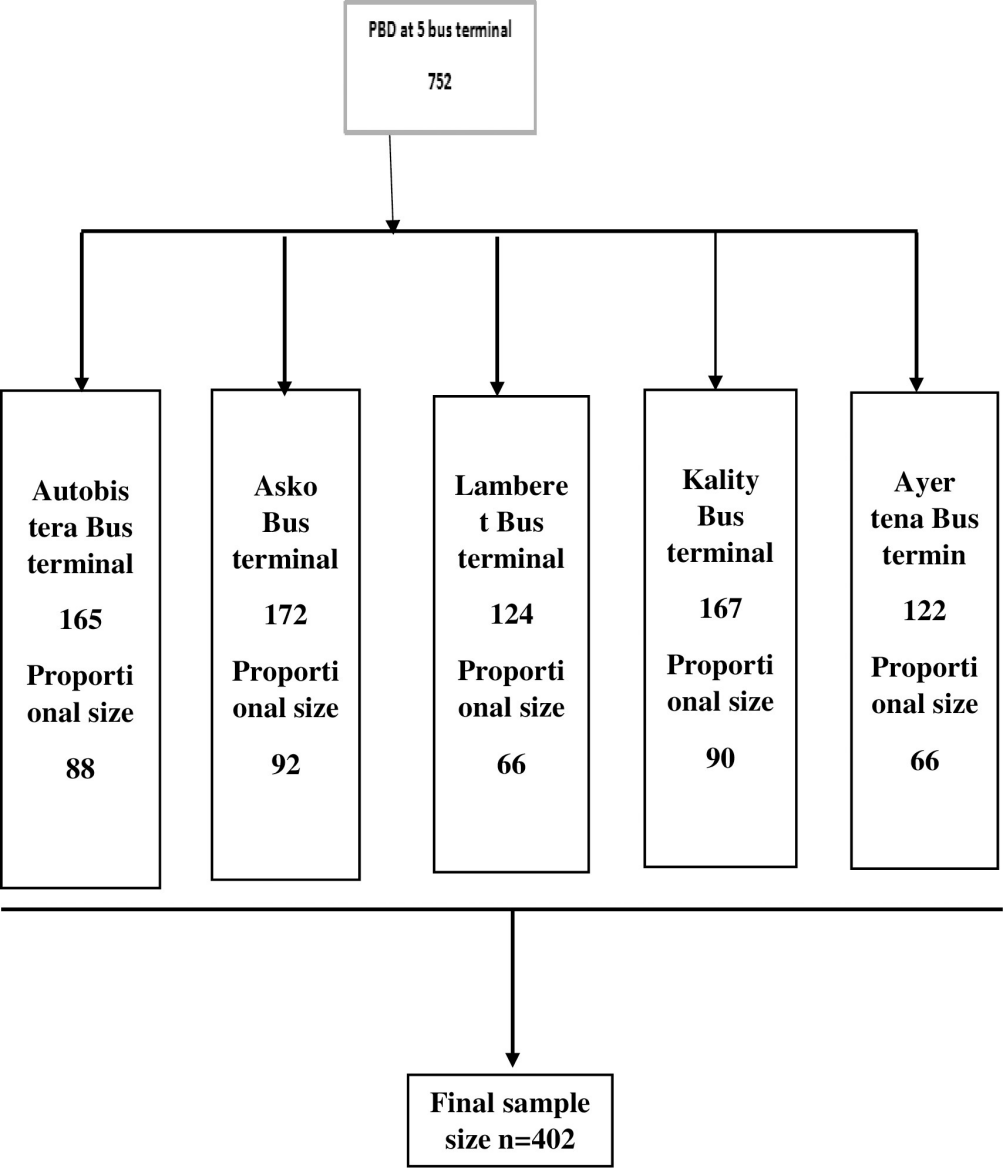
Sample size proportional allocation diagram of long-distance bus drivers in Addis bus terminals, Addis Ababa, 2021.

The list of long-distance bus drivers who have a professional driving licence and who were constantly working full time at each station was accessed from the station registered file of the private road transport union (Mahiber). From the list obtained at each station, using the population proportion formula described below, the proportional allocation of study participants for the corresponding station was estimated from the total sample size determined. Then, their identification tag was entered into SPSS software and then a computer-generated simple random sampling technique was employed to select the calculated number of participants at each station as diagrammatically expressed in (Fig 1).

### Data collection procedures and tools

A structured questionnaire was adapted based on the STEPWISE approach to the NCD study and was translated into the local language, Amharic. This study applied the face-to-face interview method for the data collection purpose in part 1 of socio-demographic, behavioral knowledge, relevant medical history, and part 2 approach to physical measurements of blood pressure and body mass index [36].

After an identification tag was given to those sampled, the day and study time were arranged at their own convenient time. Data collection was performed in a selected room of the station collaboratively arranged for this purpose. Before measuring BP, each driver was asked about alcohol, caffeine intake, chat chewing, smoking, and stressful conditions within two hours and rested for 30 minutes.

Their BP was taken 5 minutes apart two times in a sitting position with non-dominant harm using a digital omicron blood pressure apparatus, and then the average of the last two measurements was considered to determine BP status. The new 2021 ACC/AHA and International Journal of HTN guidelines were used to report participants as having HTN. According to ACC/AHA guideline, an individual with SBP greater than 140 mmHg and DBP of 90 mmHg are hypertensive [37].

### Data quality control and management

The pretest of the questionnaire was done on 5% of the calculated sample size among 20 long-distance bus drivers at the bus terminal in Sululta town, Finfine special zone. The result was not included in the final result of the study. Five data collectors and one supervisor whose profession was a Bachelor of Science in nursing were recruited and one-day training was given about the tool and data collection. Then the data collectors were deployed to the data collection after they came up with a consensus on the tool. To minimize inconvenience, the primary investigator of the study and the supervisor critically followed the data collection process.

The quality of data was assured by applying a properly designed data collection tool. Before the actual data collection, the reliability and internal consistency of the questionnaire were pretested and using the (SPSS) scale of reliability test, Cronbach’s alpha value of the questionnaires was 0.8 all over the questionnaire, 0.73 knowledge scale and 0.62 for behavioral characteristics of the questionnaire. In addition to this, training was given to data collectors and supervisors about proper categorization and coding of the questions. Finally, the data collectors were closely followed by the supervisor and the principal investigator.

### Data processing and analysis

Data entry was performed using epi data version 4.6, cleaned, checked, and exported to IBM Statistical Package for Social Science (SPSS) version 26. Descriptive data presenting such as frequency, percentage, and charts were used to describe socio-demographics, level of knowledge, behavioral characteristics, and the magnitude of the problem. Binary logistic regression analysis was used to determine associated factors for undiagnosed hypertension. A variable with a P-value of < 0.25 was considered a candidate for multivariable analysis. Multi-collinearity between independent variables and other assumptions of binary logistic regression was checked. Multivariable analysis was done using stepwise binary logistic regression to assess for independent predictors of undiagnosed hypertension. Every analysis was considered significant at a p-value of less than 0.05 with a two-tailed probability distribution.

### Ethical consideration

Before data collection, ethical clearance for the study was obtained from St. Paul Hospital Millennium Medical College Institutional review boards (IRBs) with the reference number 5714/14. In addition, a support letter was requested from the MoT. Informed consent was taken by explaining the study title, purpose, procedure, and duration. The possible risks and benefits of the study were clearly explained to the participants. All participants’ information was kept confidential and anonymous by excluding their names or any other identifying information from the tool.

They were also informed that they have the full right to refuse to participate and/or withdraw from the study at any time if they had any difficulty. All participants were educated about the implications of having high BP and were informed of their BP readings. The information gathered was kept confidential up to data insertion, after which it was held for some amount of time before being removed to prevent it from ending up in the hands of other researchers who might misuse it. An individual with elevated BP was referred using a prepared referral form to the nearest health facility to seek immediate medical attention.

## Results

### Socio-demographic characteristics of study participants

In this study, from a total of 402 study candidates, 391 participated to complete the questionnaire and physical measurements correctly. This gives a response rate of 97.2%. Of all participants 391 respondents, 100% were male drivers. Accordingly, the mean age of the participants was 41.02 (S.D of 8.639) years, and the age group of less than 40 years represents the highest group which is 52.7%. A large proportion of participants (76.7%) were married. The majority of participants, i.e., 41.2% and 40.9%, are Orthodox and Protestant religious followers, respectively. A great percentage of participants, 79%, earn less than 5250 ETB. A higher proportion of participants (52.4%) achieved a license or certificate level of education. Driving experience indicates that 45.3% served less than 10 years. Table 2 indicates the socio-economic characteristics of the study participants.

**Table 2:** Socio-demographic characteristics of long-distance bus drivers, Addis Ababa bus terminals, Ethiopia, 2022 (n = 391).

Variables	Categories	Frequency	Percentage
Age	<29	18	4.6%
	30–34	82	21%
	35–39	106	27.1%
	40–44	47	12%
	45–49	77	19.7%
	50–54	36	9.2%
	>55	15	6.4%
Marital status	Single	65	16.60%
	Married	300	76.70%
	Widowed	26	6.6%
Religious	Orthodox	161	41.20%
	Muslim	27	6.90%
	Catholic	26	6.60%
	Protestant	160	40.90%
	Others(*)	17	4.30%
Educational level	High school	45	11.50%
	Certificate	205	52.40%
	Diploma	110	28.10%
	Degree	31	7.90%
Experience	<3 years	23	5.9%
	3–5 years	26	6.6%
	6–10 years	128	32.7%
	>10 years	214	54.7%
Income	<5250ETB	309	79.0%
	>5251	82	21.0%

* “Waqefata and Traditional beliefs”

### Knowledge about hypertension

To measure the level of knowledge of HTN, a score using percentage was awarded based on the 22 questions each question getting 3 points. The level of knowledge was given based on the mean value they scored and the mean value of the level of knowledge was 41.46. A score above the mean value was labelled a good knowledge and a score below the mean value was labelled a poor knowledge.

Of the total participants, 18.7%, 23.8%, 27.1%, 18.9, 21.5%, and 27.9% had a poor level of knowledge about the cause, symptoms, prevention, treatment, and complications of hypertension respectively. Overall, 55.8% and 44.2% have good and poor knowledge of hypertension, respectively.

Of the total respondents, 29.2% were smokers and 45% of them were smoking at least 2–3 days per week regularly. Most (67.5%) of them reported that they were smoking 2–5 cigarettes at a time. Regarding hours of driving per day, 54.0% of them were driving more than 9 hours per day regularly. During their long trip, 62.9% of them had no habit of interrupting for a break.

Concerning eating habits, it is reported by this study that only 17.6% have the habit of eating fruit regularly and 90.8% of them used to eat vegetables regularly. Of the study participants, 4.1% of drivers have the habit of eating their meals while driving regularly and 34.5% of drivers consumed a high-calorie diet daily. Of the study participants, 30.7% performed regular physical exercise and 38.9% of them were performing a jogging type of exercise. Half of the participants were performing regularly greater than 5 days per week (Table 3).

**Table 3:** Behavioral characteristics of long-distance bus drivers, Addis Ababa terminals, Ethiopia, 2022 (n = 391).

Variables	Categories	Frequency	Percentage
**Smoking**	Yes	114	29.20%
	No	277	70.80%
**Regular physical exercise**	Yes	271	69.30%
	No	120	30.70%
**Eating vegetable regularly**	Yes	355	90.80%
	No	36	9.20%
**Frequency of eating a high-calorie diet**	Daily	135	34.50%
	Occasionally	172	44.00%
	Sometimes	84	21.50%
**Chewing chat**	Yes	264	67.50%
	No	127	32.50%
**Frequency of eating while driving**	Daily	16	4.10%
	Occasionally	97	24.80%
	Sometimes	137	35.00%
	Never	141	36.10%
**Frequency of eating under stressful conditions**	Daily	36	9.20%
	Occasionally	143	36.60%
	Sometimes	212	54.20%
**Hours of driving**	<9hours	180	46.0%
	>9hours	211	54.0%
**Regular eating of the fruit**	Yes	69	17.60%
	No	322	82.40%
**The regular habit of interrupting while driving**	Yes	145	37.10%
	No	246	62.90%

### History of co-morbidity

Of the 391 respondents, 24.0% of them had a family history of hypertension, while 2.6% of the participants did not know about their family history of hypertension. Comorbidities with Diabetes Mellitus accounted for 23 (5.9%) of all participants and 4.6% were previously told to have a cardiac problem. (Table 4).

**Table 4:** Clinical-related characteristics of long-distance bus drivers, Addis Ababa terminals, Ethiopia, 2022 (n = 391).

Variables	Categories	Frequency	Percentage
**Diabetes mellitus**	Yes	23	5.9%
	No	368	94.1%
**Cardiac problem**	Yes	16	4.1%
	No	375	95.9%
**Family history of hypertension**	Yes	94	24.0%
	No	287	73.4%
	Don’t know	10	2.6%

### Body mass index

**Fig 2:**
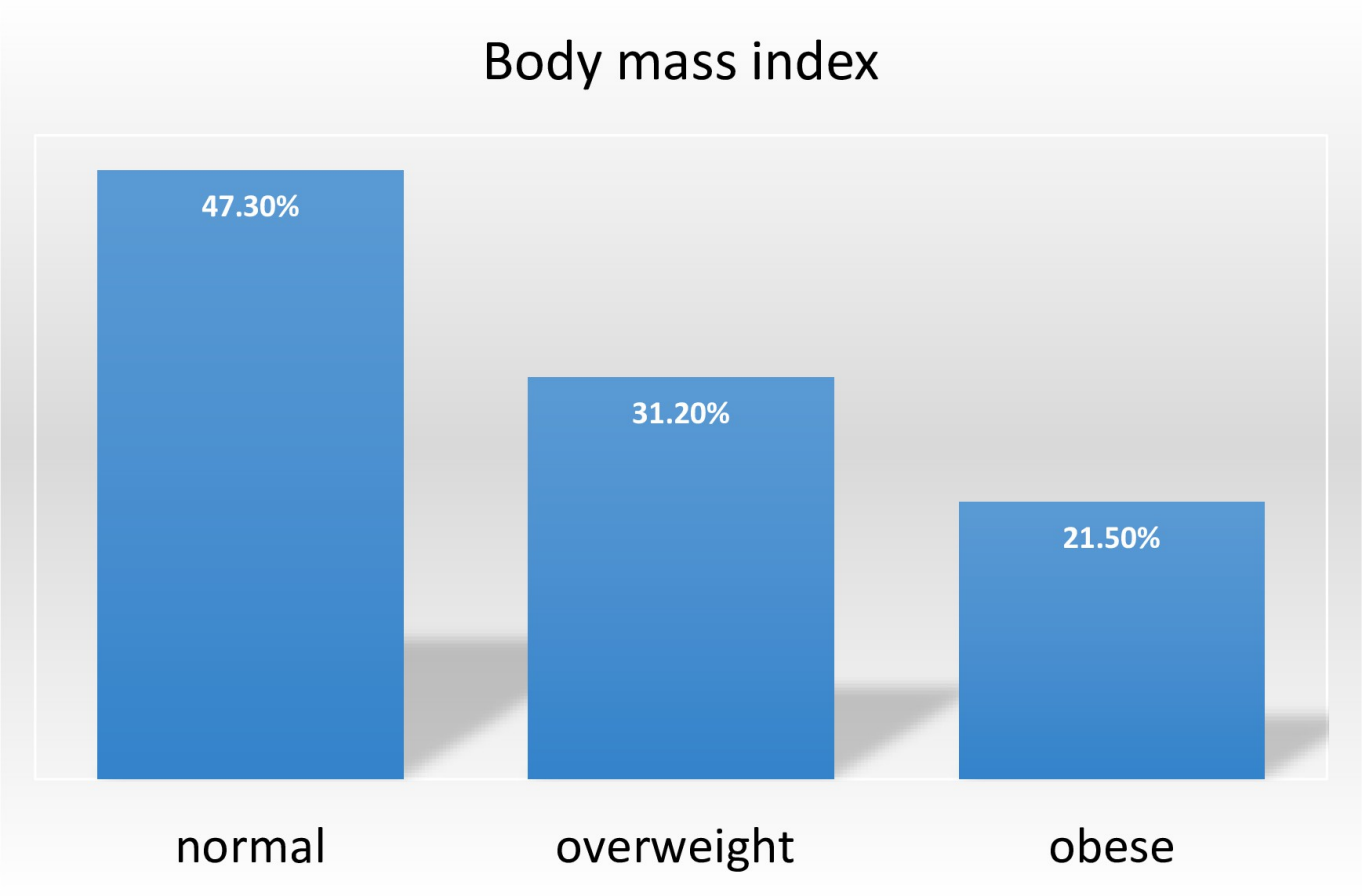
Body mass index of long-distance bus drivers in Addis Ababa terminals 2021.

Concerning BMI levels, 31.2% of the participants were overweight, while 21.5% of them were obese (Fig 2).

### Prevalence of undiagnosed hypertension

**Fig 3:**
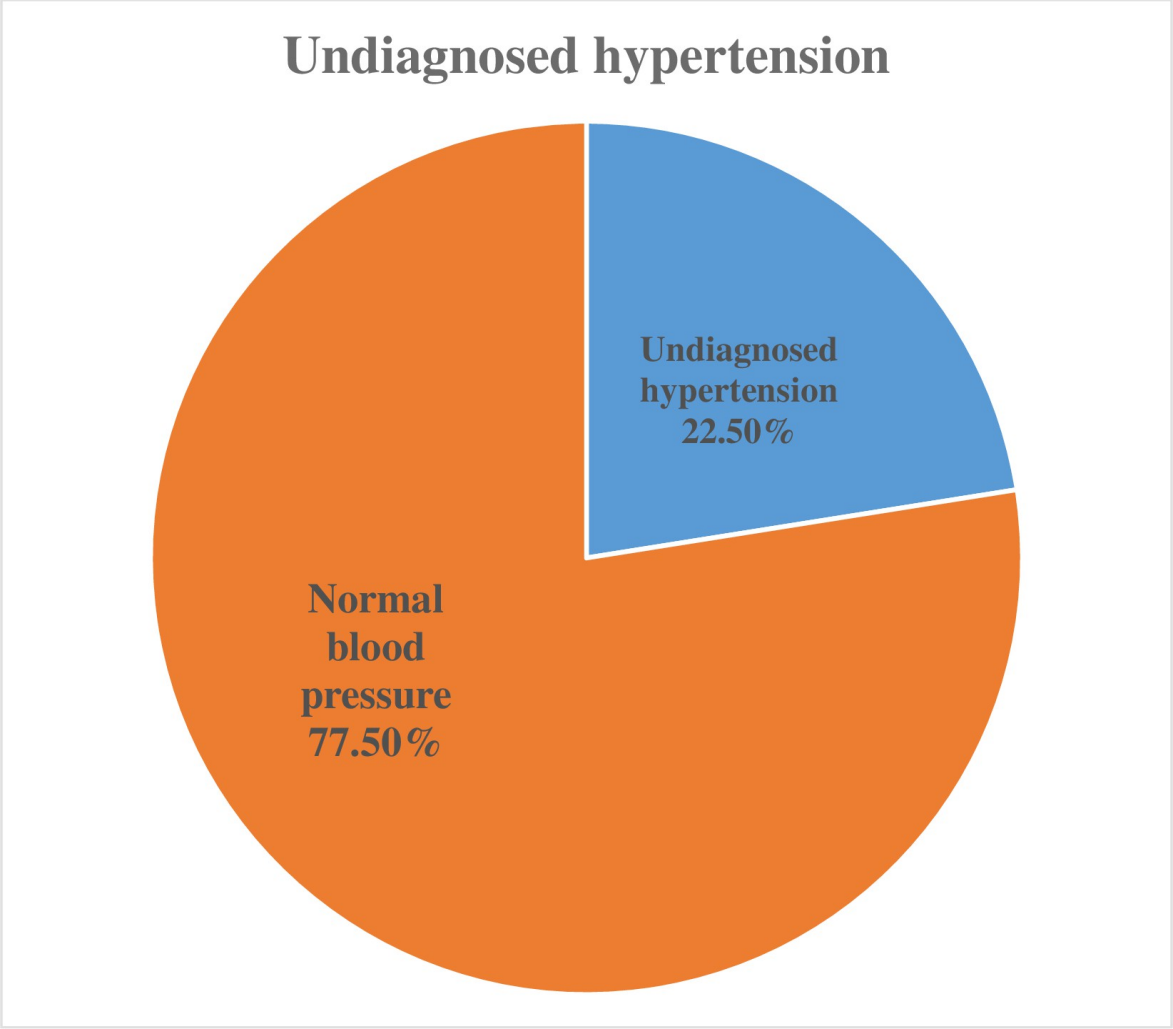
Prevalence of undiagnosed hypertension among long-distance bus drivers in a selected bus of Addis Ababa: 2022.

**Fig 4:**
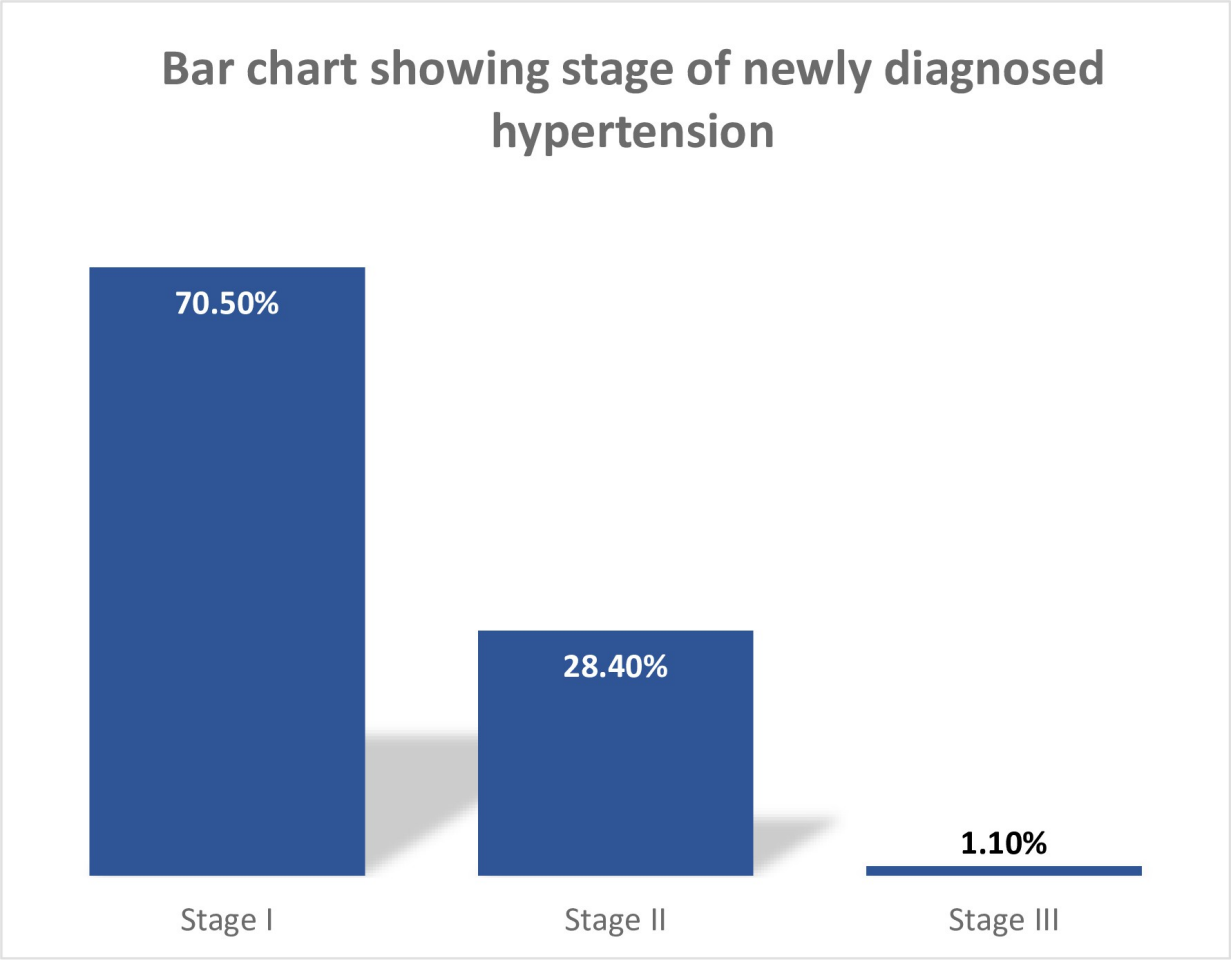
Frequency distribution of stages of newly diagnosed hypertension among long-distance bus drivers in selected bus terminals of Addis Ababa: 2022.

The prevalence of undiagnosed hypertension in this study was 22.5% (CI: 18.7%, 26.6%) and out of 391 participants, 298 had normal blood pressure. The remaining 7 had elevated blood pressure at the first measurement, however, their blood pressure level decreased to normal after the second and third blood pressure checkups (as shown in Fig 3). Among those hypertensive individuals, 71.5% have stage I hypertension, 28.74% have stage II hypertension, and 1.1% have severe HTN (as shown in Fig 4).

### Risk factors for undiagnosed hypertension

On bivariable analysis of all variables, age groups, monthly income, marital status, knowledge about HTN, smoking, chewing chat, alcohol intake, eating fruit regularly, eating while driving, hours of driving per day, BMI, regular physical exercise, and interruption during driving showed significant statistical association with undiagnosed HTN. (Shown in Table 5).

**Table 5:** Factors identified on binary logistic regression analysis with undiagnosed HTN among long-distance bus drivers in Addis Ababa terminals, 2021.

*Variables*	*Categories*	*Undiagnosed HTN (n = 88)*		*p-value*	*COR*	*95% CI*
		*Yes*	*No*			
*Age*	<29	5(27.8%)	13(72.2%)	0.001*	**1**	**1**
	30–34	12(14.6%)	70(85.4%)	0.187	2.244	(0.676,7.445)
	35–39	15(14.2%)	91(85.8%)	0.155	2.333	(0.726,7.496)
	40–44	10(21.3%)	37(78.7%)	0.579	1.423	(0.409,4.946)
	45–49	18(23.4%)	59(76.6%)	0.695	1.261	(0.396,4.016)
	50–54	16(44.4%)	20(55.6%)	0.241	0.481	(0.141,1.633)
	>55	12(48.0%%)	13(52%)	0.185	0.417	(0.114,1.523)
*Monthly income*	<5200	55(17.8%)	254(82.2%)		**1**	**1**
	>5201	33(40.2%)	49(59.8%)	0.000*	0.322	(0.189,5.46)
*Marital status*	Single	7(10.8%)	58(89.2%)	0.014*	**1**	**1**
	Married	71(23.7%)	229(76.3%)	0.026*	0.389	(0.170,0.891)
	Divorced	10(38.5%)	16(61.5%)	0.004*	0.193	(0.063,0.588)
*Knowledge level*	Good knowledge	26(15.0%)	147(85.0%)	0.000*	**1**	**1**
	Poor knowledge	62(28.4%)	156(71.6%)	0.002*	2.247	(1.349,3.743)
*Regular chewing chat*	Yes	53(41.7%)	74(58.3%)	0.000*	4.686	(2.84,7.73)
	No	35(13.3%)	229(86.7%)		**1**	**1**
*Smoking*	Yes	42(36.8%)	72(63.2%)	0.000*	0.341	(0.208,0.560)
	No	46(16.6%)	231(83.4%)		**1**	**1**
*Alcohol consumption*	Yes	74(31.5%)	161(68.5%)	0.000*	4.662	(2.523,8.616)
	No	14(9.0%)	142(91.0%)		**1**	**1**
*Regular fruit-eating*	Yes	23(33.3%)	46(66.7%)	0.019*	0.506	(0.286,0.894)
	No	65(20.2%)	257(79.8%)		**1**	**1**
*Eating while driving*	Daily	8(50.0%)	8(50.0%)	0.005*	0.216	(0.074,0.629)
	Occasionally	22(22.7%)	75(77.3%)	0.347	0.735	(0.38,1.39)
	Sometimes	33(24.1%)	104(75.9%)	0.194	0.679	(0.37,1.21)
	Never	25(17.7%)	116(82.3%)	0.044	**1**	**1**
*Hours of driving*	<9 hours	28(13.3%)	183(86.7%)	0.000*	**1**	**1**
	>9 hours	60(33.3%)	120(66.7%)	0.467	0.781	(0.401,1.521)
*Physical exercise*	Yes	59(49.2%)	61(50.8%)	0.000*	0.124	(0.073,0.210)
	No	29(10.7%)	242(89.3%)		**1**	**1**
*Interruption of rest while driving*	Yes	38(15.4%)	208(84.6%)	0.000*	0.347	(0.213,0.565)
	No	50(34.5%)	95(65.5%)		**1**	**1**
*BMI level*	Normal	60(32.3%)	126(67.7%)	0.000	**1**	**1**
	Overweight	16(12.6%)	111(87.4%)	0.000*	3.304	(1.799,6.066)
	Obese	12(15.4%)	66(84.6%)	0.006*	2.619	(1.317,5.209)

* P-Value < 0.25 was considered as statistically association OR: odds ratio; CI: Confidence interval; 1: reference.

In a stepwise final multivariable logistic regression analysis; the level of knowledge of HTN, regular physical exercise, body mass index, hours of driving per day, alcohol drinking, and chewing chat maintained a significant association to predict undiagnosed HTN among study participants.

Concerning the level of knowledge of HTN, bus drivers who have poor knowledge were 2.00 times (AOR: 2.00; 95% CI 1.08, 3.70) more likely than drivers with good knowledge about HTN.

Bus drivers who work greater than 9 hours a day were 2.50 times (AOR: 2.50; 95% CI 1.37, 4.56) more likely than 7 hours per day. Drivers who have a habit of chewing chat were 2.61 times (AOR: 2.61; 95% CI: 1.44, 4.73) more likely to have undiagnosed HTN than those who were non-chewers.

Participants who drank alcohol regularly were 3.46 times (AOR: 3.46; 95% CI: 1.70, 7.05) riskier than those who did not drink alcohol to have undiagnosed HTN. Performing regular physical exercise decreased the likelihood of having undiagnosed HTN by 84.0% (AOR: 0.16, 95%; CI: 0.09, 0.29). Bus drivers who were overweight were 3.14 times (AOR: 3.14, 95%; CI 1.54, 6.42) more likely, and obese drivers were 3.21 times (AOR: 3.21, 95%; CI 1.35, 7.61) more likely to have undiagnosed HTN than participants with normal BMI (Table 6).

**Table 6:** Factors identified on multivariable logistic regression analysis with undiagnosed HTN among long-distance bus drivers in Addis Ababa terminals, 2022.

Variables	Categories	Undiagnosed HTN		COR	95% CI	AOR	95% CI	P-value
		Yes	No					
Knowledge level	Good knowledge	26(15.0%)	147(85.0%)	**1**	**1**	**1**	**1 **	**1**
	Poor knowledge	62(28.4%)	156(71.6%)	2.247	(1.34,3.74)	2.003	(1.08,3.70)	0.027**
Alcohol consumption	Yes	74(31.5%)	161(68.5%)	4.662	(2.52,8.61)	3.46	(1.70,7.05)	0.001**
	No	14(9.0%)	142(91.0%)	**1**	**1**	**1**	**1**	**1**
Regular chewing chat	Yes	53(41.7%)	74(58.3%)	4.686	(2.84,7.73)	2.61	(1.44,4.76)	0.001**
	No	35(13.3%)	229(86.7%)	**1**	**1**	**1**	**1**	**1**
Hours of driving	<9 hours	28(13.3%)	183(86.7%)	**1**	**1**	**1**	**1 **	**1**
	>9 hours	60(33.3%)	120(66.7%)	3.268	(1.974,5.41)	2.50	(1.37,4.56)	0.003**
Physical exercise	Yes	29(10.7%)	242(89.3%)	0.124	(0.07,0.21)	0.161	(0.09,0.29)	0.000**
	No	59(49.2%)	61(50.8%)	**1**	**1**	**1**	** 1**	**1**
BMI level	Normal	60(32.3%)	126(67.7%)	**1**	**1**	**1**	** 1**	0.002**
	Overweight	16(12.6%)	111(87.4%)	3.304	(1.79,6.06)	3.14	(1.54,6.42)	0.008**
	Obese	12(15.4%)	66(84.6%)	2.619	(1.31,5.20)	3.21	(1.35,7.61)	0.003**

** P-Value < 0.05 was considered a statistically significant association OR: odds ratio; CI: Confidence interval; 1: reference.

## Discussion

This facility-based cross-sectional study was performed to assess the prevalence of undiagnosed HTN and associated factors among long-distance bus drivers. Overall, the prevalence of undiagnosed HTN was 22.5%, with a 95% CI (18.2–26.9). The prevalence of undiagnosed HTN in this study is in line with similar studies conducted in Karnataka, India, and Mojo terminal, which was 23.2% and 20%, respectively [38, 39].

The finding of this study indicates that long-distance bus drivers have found a higher prevalence of undiagnosed HTN when compared with the general population in different parts of Ethiopia. Studies in Hawasa, Gulale sub-city and Dano district indicate 13.25%, 12.3%, and 14.6% of undiagnosed HTN respectively [16, 30, 31]. This difference from the general population may be due to the nature of the occupation. Long-distance driving is largely sedentary, psychologically, and physically stressful, which could in turn cause obesity. In this study, obesity was a predictor of HTN.

The results of this study showed a higher prevalence of undiagnosed HTN when compared to a similar study conducted in Bangalore city, India, which was 16.0%. The discrepancy may be due to the difference in sample size, operational difference, measuring devices used and the number of facilities. The study in Bengaluru was performed at a single facility and used 60 bus drivers [23].

On the other hand, the proportion of undiagnosed HTN in this study is lower when compared to similar studies conducted in Ghana, Turkey, North Kerala, India, Saudi Arabia, and Nigeria which were 38.7%, 31.4%, 41.3%, 38.7%, and 33.5% respectively [40–43]. The difference could be owed to the type of diet and sociodemographic, cultural and genetic differences. High consumption of potassium and sodium-rich foods among West African countries such as Ghana and Nigeria could result in higher prevalence [44]. In addition, genetic predisposition made the greatest odds of HTN among Southeast Asian countries such as India [45].

In line with the hypothesis, this study identified knowledge of HTN, behavior, overweight, and obesity as associated factors for undiagnosed HTN. In this study, bus drivers with poor knowledge about HTN were twice as likely to have undiagnosed HTN when compared with those who had good knowledge. This association is plausible, as a majority of long-distance drivers achieved a lower educational status which could have resulted in low awareness. This implies that most bus drivers are unaware of the asymptomatic nature of HTN, its cause, prevention mode, and treatment methods and that it can lead to complications if untreated. Individuals with good awareness of HTN might have a better healthy lifestyle and health-seeking behavior, and less vulnerability to HTN risk behaviors. This association is supported by other numerous studies in Bahir Dar, Dano district, Hawasa, and India [23, 30, 31, 46].

The result of the data showed that chewing chat was one of the predictors of undiagnosed HTN. During the interview, the bus drivers practiced chewing chat to prevent daytime sleeping while on their journey. People who chew chat are less likely to notice the early symptoms of HTN because it makes them asymptomatic and prevents them from visiting a health facility. In addition, the habit may influence them economically compromised to afford the payment for health facility visits. This result is in line with a similar study conducted in Hawasa, Ethiopia, to find chewing chat as a risk factor for undiagnosed HTN [30].

Respondents who consumed alcohol regularly were nearly three times more likely to have undiagnosed HTN than those who did not consume alcohol. This finding is supported by another cross-sectional study in Brazil and Ghana [33, 47]. Alcohol causes HTN by inducing the secretion of cortisol, which further increases angiotensin II production through the RAAS and leads to elevated BP [48].

Most of our study participants responded that they work for several hours to gain additional income. Specifically, participants who work greater than 9 hours regularly per day were two times more likely to have undiagnosed HTN than those who work less than 9 hours. The longer duration of diving hours may result in hypertension due to more hours of sitting, a sedentary lifestyle, and physical inactivity [49]. The prolonged driving hour also increases stress levels and constant exposure to a noisy environment. This association is in agreement with the studies conducted in Mojo, Ethiopia, and Australia [39, 50].

This study is in harmony with other similar studies in Hawasa, India, Nigeria, Ghana, and elsewhere in considering increased BMI as a predictor of undiagnosed HTN [23, 33, 39, 40]. Participants who were obese and overweight were shown to have higher odds of undiagnosed HTN. Obesity among bus drivers could be attributed to regular high-calorie consumption, alcohol consumption, and lack of physical exercise.

Obesity and overweight increase stroke volume and cardiac output in addition to the vasoconstriction effect and an increased pharyngeal soft tissue that causes obstructive sleep apnea during sleeping [51]. Therefore, efforts to control unhealthy weight gain should be encouraged for controlling obesity to prevent HTN.

Performing regular physical exercise decreased the odds of having undiagnosed HTN. Long-distance drivers lack the appropriate time to perform physical activity as they rush to finish their mandate, and they end up sitting for long hours without activity. Apart from their long journey, during their vacation days, bus drivers even use their buses rather than walking for intracity short distance travelling. This association is supported by scientific evidence that physical exercise prevents elevated BP by reducing the stiffening and narrowing of the blood vessel lumen [52]. Moreover, the result is consistent with other studies conducted in Taipei, Ghana, and Nigeria [23, 53, 54].

In general, long-distance bus drivers are at a higher risk of burden from unscreened HTN. They are suffering from chronic undiagnosed hypertension. This is because of a lack of adequate awareness, behavioral, occupational, and increased BMI. This could create a great burden of mortality and morbidity caused by undiagnosed HTN. Therefore, this study highlights, that further research is needed to investigate other possible causes for undiagnosed HTN among long-distance bus drivers.

## Conclusion

The proportion of undiagnosed HTN in this study among long-distance bus drivers is significant (22.5%). Identified factors which increased the odds of undiagnosed HTN were lack of adequate knowledge of HTN, behavioral factors such as regular alcohol and chat consumption, long duration of working hours, overweight, and obesity. On the other hand, having the habit of performing regular physical exercise decreased the odds of having undiagnosed HTN. This study also identified the major co-morbidities among this population were overweight and obesity.

## Recommendation

The result of this study underscores the necessity and high demand for preventive strategies of HTN among long-distance bus drivers. The investigator respectfully recommends the following tips for the respective responsible organizations/bodies.

FMoH in collaboration with transportation authorities should develop guidelines and policies for regular and periodic blood pressure checkups for all passengers carrying long-distance drivers. If possible, the Ministry of Health in collaboration with the Ministry of Transport should open onsite clinics at each bus terminal. Based on the results of our study, the Ministry of Transport should put into effective action the policy of banning khat.

Policymakers should enable the enactment of national physical activity recommendations among long-distance bus drivers. In addition, different transport unions are better educated about preventive strategies such as working on shift for longer hours drivers can decrease the likelihood of HTN.

It is also warranted for researchers, specifically cardiac specialists, to conduct prospective cohort studies to determine cardio-metabolic profiling among obese bus drivers. Applying part III of the WHO approach to NCD. Based on the identified factors in this study, cardiovascular nurses and specialists can develop a predictive tool for hypertension occurrence among long-distance bus drivers.

## Strength and limitations of the study

This study is the first of its type in Ethiopia among long-distance bus drivers. In addition, it is performed in multi-facility, the study was conducted at all five bus terminals found in Addis Ababa. This will increase the representativeness of the finding.

Despite these strengths, the limitation of this study was the inability to measure the abdominal circumference, it is better than BMI to predict HTN. It was beyond the scope of this study to apply part III of the WHO approach to measure biochemical components such as blood glucose level, serum cholesterol, etc. by the approach was also drawn back.

## Supporting information

S1 Annex (DOCX)

## Abbreviations/ acronyms

ACC: American College of Cardiology
AHA: American Heart Association
BMI: Body Mass Index
BP: Blood Pressure
DBP: Diastolic blood pressure
DM: Diabetes mellitus
FMoH: Federal Ministry of Health
PIS: Plain insight
HTN: Hypertension
mmHg: millimeter of mercury
MoT: Ministry of Transport
NCD: Non-communicable disease
SBP: Systolic blood pressure
SPHMMC: Saint Paul’s Hospital Millennium Medical College
SPSS: Statistical package for social science
WHO: World Health Organization

## References

[1] Hermida R.C., et al., New perspectives on the definition, diagnosis, and treatment of true arterial hypertension. Expert Opinion on Pharmacotherapy, 2020. 21(10): p. 1167–1178. doi: 10.1080/14656566.2020.1746274 32543325

[2] Organization., W.H. Hyperetension. 2021 2021b, August 25; Available from: https://Www.Who.Int/Health

[3] He J. and Whelton P.K., Elevated systolic blood pressure and risk of cardiovascular and renal disease: overview of evidence from observational epidemiologic studies and randomized controlled trials. American heart journal, 1999. 138(3): p. S211–S219. doi: 10.1016/s0002-8703(99)70312-1 10467215

[4] Wang H., et al., Blood transcriptome profiling as potential biomarkers of suboptimal health status: potential utility of novel biomarkers for predictive, preventive, and personalized medicine strategy. EPMA Journal, 2021. 12(2): p. 103–115. doi: 10.1007/s13167-021-00238-1 34194583 PMC8192624

[5] Cesani M.F., et al., High blood pressure in children and adolescents from urban peripheral areas of La Plata, Argentina. Revista Salud Uninorte, 2020. 36(1): p. 62–80.

[6] organization W.H., More than 700 million people with untreated hypertension. 2021.

[7] John L., Flin R., and Mearns K., Bus driver well-being review: 50 years of research. Transportation research part F: traffic psychology and behaviour, 2006. 9(2): p. 89–114.

[8] Cheung C.K., et al., Cardiovascular risk in bus drivers. Hong Kong Med J, 2020. 26(5): p. 451–6. doi: 10.12809/hkmj198087 33089795

[9] Chockalingam A., Impact of world hypertension day. Canadian journal of cardiology, 2007. 23(7): p. 517–519. doi: 10.1016/s0828-282x(07)70795-x 17534457 PMC2650754

[10] WHO W., A global brief on hypertension: Silent killer, global public health crisis. 2013.

[11] AlMarzooq A.M., Emergency Department Nurses’ Knowledge Regarding Triage. International Journal of Nursing, 2020. 7(2): p. 29–44.

[12] Zhou B., et al., Global epidemiology, health burden and effective interventions for elevated blood pressure and hypertension. Nature Reviews Cardiology, 2021: p. 1–18. 10.1038/s41569-021-00559-8 PMC8162166 34050340

[13] Mendis S., Bettcher D., and Branca F., World Health Organization Global Status Report on Noncommunicable Diseases. 2014. 2014. 10.1161/STROKEAHA.115.008097 25873596

[14] Badego B., Yoseph A., and Astatkie A., Prevalence and risk factors of hypertension among civil servants in Sidama Zone, south Ethiopia. PloS one, 2020. 15(6): p. e0234485. doi: 10.1371/journal.pone.0234485 32525916 PMC7289366

[15] Abebe S. and Yallew W.W., Prevalence of hypertension among adult outpatient clients in hospitals and its associated factors in Addis Ababa, Ethiopia: a hospital based cross-sectional study. BMC research notes, 2019. 12(1): p. 1–6. 30764864 10.1186/s13104-019-4127-1 PMC6376725

[16] Getachew F., Dirar A., and Solomon D., Prevalence of undiagnosed hypertension and associated factors among residents in Gulele Sub-City, Addis Ababa, Ethiopia. J Community Med Health Educ, 2018. 8(590): p. 2161–0711.1000590.

[17] Zeru A.B. and Muluneh M.A., Admission and Inpatient Mortality of Hypertension Complications in Addis Ababa. Integrated Blood Pressure Control, 2020. 13: p. 103. doi: 10.2147/IBPC.S268184 32982396 PMC7509485

[18] Bokaba M., Modjadji P., and Mokwena K.E.. Undiagnosed Hypertension in a Workplace: The Case of a Logistics Company in Gauteng, South Africa. in Healthcare. 2021. Multidisciplinary Digital Publishing Institute. 10.3390/healthcare9080964 PMC8394589 34442101

[19] Hedberg G.E., et al., Risk indicators of ischemic heart disease among male professional drivers in Sweden. Scandinavian journal of work, environment & health, 1993: p. 326–333. doi: 10.5271/sjweh.1467 8296181

[20] Wang P.D. and Lin R.S., Coronary heart disease risk factors in urban bus drivers. Public health, 2001. 115(4): p. 261–264. doi: 10.1038/sj/ph/1900778 11464297

[21] Krishnamoorthy Y., Sarveswaran G., and Sakthivel M., Prevalence of hypertension among professional drivers: Evidence from 2000 to 2017—A systematic review and meta-analysis. Journal of postgraduate medicine, 2020. 66(2): p. 81. doi: 10.4103/jpgm.JPGM_297_19 32134003 PMC7239404

[22] Nimi, B.M., et al., Prevalence of Undiagnosed Hypertension among the Hypertensives Living in the City of Boma. Republic Democratic Republic of the Congo.

[23] Pushpa K. and Kanchana R., Comparison of waist-hip ratio, prehypertension, and hypertension in young male bus drivers and non-drivers of Bengaluru city. National Journal of Physiology, Pharmacy and Pharmacology, 2019. 9(1): p. 90–94.

[24] Akinremi A., A Survey of Undiagnosed Hypertension among Market Traders in Suva, Fiji Islands. African Journal of Biomedical Research, 2020. 23(2): p. 193–197.

[25] Mbakwem A.C., et al., Prevalence of cardiometabolic risk factors among professional male long-distance bus drivers in Lagos, south-west Nigeria: a cross-sectional study. Cardiovascular journal of Africa, 2018. 29(2): p. 106–114. doi: 10.5830/CVJA-2018-006 29457826 PMC6008896

[26] Winkleby M.A., et al., Excess risk of sickness and disease in bus drivers: a review and synthesis of epidemiological studies. International journal of epidemiology, 1988. 17(2): p. 255–262. doi: 10.1093/ije/17.2.255 3042649

[27] Miao Q., et al., Sudden death from ischemic heart disease while driving: cardiac pathology, clinical characteristics, and countermeasures. Medical Science Monitor: International Medical Journal of Experimental and Clinical Research, 2021. 27: p. e929212–1. doi: 10.12659/MSM.929212 33495433 PMC7847085

[28] Tüchsen F., et al., Stroke among male professional drivers in Denmark, 1994–2003. Occupational and Environmental Medicine, 2006. 63(7): p. 456–460. doi: 10.1136/oem.2005.025718 16735481 PMC2092514

[29] van de Vijver S., et al., Status report on hypertension in Africa-Consultative review for the 6th Session of the African Union Conference of Ministers of Health on NCD’s. Pan African Medical Journal, 2014. 16(1). 10.11604/pamj.2013.16.38.3100 PMC3932118 24570798

[30] Wachamo D., Geleta D., and Woldesemayat E.M., Undiagnosed Hypertension and Associated Factors Among Adults in Hawela Tula Sub-City, Hawassa, Southern Ethiopia: A Community-Based Cross-Sectional Study. Risk Management and Healthcare Policy, 2020. 13: p. 2169. doi: 10.2147/RMHP.S276955 33116995 PMC7573300

[31] Regea F., Distribution of risks and Factors Associated with Unscreened Hypertension among Adults Living in Rural of Dano District, West Shewa, Oromia, Ethiopia, 2020: Community-based cross-sectional study. 2021.

[32] organization, w.h. Ethiopia sets to improve hypertension prevention and control at primary health care level. 2020 [cited 2021 December 12]; Available from: https://www.afro.who.int/news/ethiopia-sets-improve-hypertension-prevention-and-control-primary-health-care-level.

[33] Anto E.O., et al., Prevalence and lifestyle-related risk factors of obesity and unrecognized hypertension among bus drivers in Ghana. Heliyon, 2020. 6(1): p. e03147. doi: 10.1016/j.heliyon.2019.e03147 32042945 PMC7002790

[34] agency E. s. Addis Ababa, Ethiopia Metro Area Population 1950–2021. 2021 [cited 2021 Nov 10]; Available from: www.macrotrends.net.

[35] map, g. Distance Between Addis Ababa and Surrounding Cities. 2021 [cited 2021 December 5]; Available from: https://www.distancefromto.net/city-addis-ababa-et.

[36] Organization W.H., WHO STEPS surveillance manual: the WHO STEPwise approach to chronic disease risk factor surveillance. 2005, World Health Organization.

[37] Greenland P. and Peterson E., The new 2017 ACC/AHA guidelines “up the pressure” on diagnosis and treatment of hypertension. Jama, 2017. 318(21): p. 2083–2084. doi: 10.1001/jama.2017.18605 29159417

[38] Joshi A.V., et al., Prevalence of hypertension and its socio demographic and occupational determinants among bus drivers in North Karnataka–A Cross sectional study. Education, 2013. 50(53): p. 14.5.

[39] Yosef T., Prevalence and associated factors of chronic non-communicable diseases among cross-country truck drivers in Ethiopia. BMC public health, 2020. 20(1): p. 1–7. 33069207 10.1186/s12889-020-09646-w PMC7568414

[40] Lakshman A., et al., Prevalence and risk factors of hypertension among male occupational bus drivers in North Kerala, South India: a cross-sectional study. International Scholarly Research Notices, 2014. 2014. doi: 10.1155/2014/318532 24971195 PMC4045462

[41] Hayran O., TAŞDEMİR M., and Hasan Hüseyin E., Hypertension and Obesity in Male Bus Drivers. Turkiye Klinikleri J Med Sci, 2009. 29(4): p. 826–32.

[42] Vincent-Onabajo G.O., Adaji J.O., and Umeonwuka C.I., Prevalence of undiagnosed hypertension among traders at a regional market in Nigeria. Annals of Medical and Health Sciences Research, 2017. 7(2): p. 97–101.

[43] Abdelmoneim I., Hearing impairment and hypertension among long distance bus drivers. Journal of family & community medicine, 2003. 10(3): p. 25. 23012034 PMC3425749

[44] Okello S., et al., Hypertension prevalence, awareness, treatment, and control and predicted 10-year CVD risk: a cross-sectional study of seven communities in East and West Africa (SevenCEWA). BMC Public Health, 2020. 20(1): p. 1–13. 33187491 10.1186/s12889-020-09829-5 PMC7666461

[45] Fei K., Racial and ethnic subgroup disparities in hypertension prevalence, New York City Health and Nutrition Examination Survey, 2013–2014. Preventing chronic disease, 2017. 14. doi: 10.5888/pcd14.160478 28427484 PMC5420441

[46] Dejenie M., Kerie S., and Reba K., Undiagnosed hypertension and associated factors among bank workers in Bahir Dar City, Northwest, Ethiopia, 2020. A cross-sectional study. PloS one, 2021. 16(5): p. e0252298. doi: 10.1371/journal.pone.0252298 34043717 PMC8158901

[47] Vieira M.C., Sperandei S., and Reis A., Physical activity overcomes the effects of cumulative work time on hypertension prevalence among Brazilian taxi drivers. The Journal of Sports Medicine and physical fitness, 2015. 27285352

[48] Husain K., Ansari R.A., and Ferder L., Alcohol-induced hypertension: Mechanism and prevention. World journal of cardiology, 2014. 6(5): p. 245. doi: 10.4330/wjc.v6.i5.245 24891935 PMC4038773

[49] Chankaramangalam M.A., et al., Factors associated with hypertension among truck drivers: a cross sectional study at a check post on a national highway in South India. International Journal of Medical Research & Health Sciences, 2017. 6(5): p. 126–129.

[50] Brodie A., et al., Australian bus drivers’ modifiable and contextual risk factors for chronic disease: A workplace study. Plos one, 2021. 16(7): p. e0255225. doi: 10.1371/journal.pone.0255225 34324584 PMC8321218

[51] Gutiérrez-Cuevas J., Santos A., and Armendariz-Borunda J., Pathophysiological molecular mechanisms of obesity: A link between MAFLD and NASH with cardiovascular diseases. International Journal of Molecular Sciences, 2021. 22(21): p. 11629. doi: 10.3390/ijms222111629 34769060 PMC8583943

[52] Mosca L., et al., National study of physician awareness and adherence to cardiovascular disease prevention guidelines. Circulation, 2005. 111(4): p. 499–510. doi: 10.1161/01.CIR.0000154568.43333.82 15687140

[53] Young D.R., et al., Sedentary behavior and cardiovascular morbidity and mortality: a science advisory from the American Heart Association. Circulation, 2016. 134(13): p. e262–e279. doi: 10.1161/CIR.0000000000000440 27528691

[54] Chen M.-J., Zheng C.-S., and Jeng H.-M., Taipei Bus Drivers’ Attitude and Intention to Control Hypertension.

