# Supplementary material for: Undiagnosed hypertension and associated factors among long-distance bus drivers in Addis Ababa terminals, Ethiopia, 2022: A cross-sectional study
Source: PLoS One. 2024 Feb 15;19(2):e0292890. doi: 10.1371/journal.pone.0292890 (PMC10868739; doi:10.1371/journal.pone.0292890)
Supplement: S1 Annex — (DOCX) [file pone.0292890.s001.docx]

ANNEXES

Questionnaires English version **(**[**1-5**](#_ENREF_1)**)**

Structured questionnaires constructed from different literatures to assess the undiagnosed HTN and associated factors among professional long distance bus drivers, in Addis Ababa cross country bus terminals, Addis Ababa, Ethiopia 2021 G.C.

**Annex 1**. Participant’s Informed Consent Form

Good morning/afternoon, my name is……………………………... I am here on behalf of **Abebaw Bires and others** for collecting data of a study titled undiagnosed HTN and associated factors in Addis Ababa terminals, Addis Ababa, Ethiopia. I am a data collector conducting a research for the partial fulfillment of the requirements for the degree of Master in Cardiovascular nurse practitioner at St. Paul hospital millennium medical college. I guarantee that your identity will be kept confidential and your involvement is completely based on volunteer participation. I will not ask for your name or other information that directly identify about you. There are no any risks anticipated for this study and no benefit or compensation will be awarded for participating in the study but the results of your response will be essential for policy makers in regards to identify problems. You have the right to participate or not to respond to the questions and physical measurements partially or fully and you have also the right to withdraw your participation at any time during interview. The questioner will last at most 5 -10 minutes. And the blood pressure measurement, height and weight measurements require at most 15 minutes. If you agree to participate, you will start the interview. Before we start, I need you to explain that I have read you this consent and you understand and freely agree to interview the quaternary.

Are you Volunteer? 1. Yes 2. No

If yes, please continue, if not stop participation of the study.

If you have any inquiry/question pleases contact: Abebaw Bires Adal

Call phone + 251 925841566 [email: abebawbires27@gmail.com]

Name of data collector: ________________ Signature: _____________ Date:

**Annex 2: English Version Questionnaire**

Dear respondents: - This questionnaire was designed on thesis titled of “Undiagnosed HTN and associated factors among long distance professional bus drivers at Addis Ababa terminals, Addis Ababa Ethiopia, 2022”. The outcome of the study will be used in order to suggest possible solutions for problems identified while conducting the study. I kindly request you to spent your precious time to tell the questionnaire and physical measurements as frank as and reasonable as possible. I inform you that, the information you provide will be consumed for research purpose only. The information you provide will also be kept confidential. Therefore, you all not expected to tell your name.

Thank you for your cooperation!

Signature ---------------------

| **Instruction:** - I kindly request you to answer each question as I read. | | |
| --- | --- | --- |
| **Part I. Socio demographic characteristics** | | |
| **No** | **Variable** | **Response** |
| 101 | What is your age in year? | -------------- years old |
| 102 | Sex | 1. Male 2. Female |
| 103 | What is your marital status? | 1. Never married 2. Married 3. Divorced 4. Widowed |
| 104 | What is your educational level? | -------------------- |
| 105 | How much is your family size? | -------------------- |
| 106 | How much is your working experience? | -------------------Months/Years |
| 107 | How much is your monthly income? | --------------------Ethiopian birr |
| **Part II. Knowledge towards HTN** | | |
| **A. Knowledge towards the causation of HTN** | | |
| 201 | Eating diet rich in salt can cause HTN. | 1. 1. Yes 2. 2. No 3. 3. Don’t know |
| 202 | Being overweight can cause HTN. | 1. Yes  2. No  3. Don’t know |
| 204 | Too much drinking of alcohol can cause HTN. | 1. Yes  2. No  3. Don’t know |
| 205 | Smoking cigarette can cause HTN. | 1. Yes 2. No 3. Don’t know |
| **B. knowledge towards the signs and symptoms of HTN** | | |
| 207 | Headache is the symptoms of HTN. | 1. Yes 2. No 3. Don’t know |
| 208 | Dizziness is the symptoms of HTN. | 1. Yes 2. No 3. Don’t know |
| 210 | Palpitation is the sign and symptoms of HTN. | 1. Yes 2. No 3. Don’t know |
| **C. Knowledge towards prevention of HTN** | | |
| 211 | Exercising regularly could prevent HTN. | 1. Yes 2. No 3. Don’t know |
| 212 | Monitoring of blood pressure is very important in order to prevent high blood pressure. | 1. Yes 2. No 3. Don’t know |
| 213 | Reducing stress level could prevent HTN. | 1. Yes 2. No 3. Don’t know |
| 214 | Eating fruits and vegetables could prevent HTN. | 1. Yes 2. No 3. Don’t know |
| 215 | Reducing the amount of salt intake could prevent HTN. | 1. Yes 2. No 3. Don’t know |
| **D. Knowledge towards complication of HTN** | | |
| 216 | HTN can cause heart diseases, if left untreated. | 1. Yes 2. No 3. Don’t know |
| 217 | HTN can cause visual impairment, if left untreated. | 1. Yes 2. No 3. Don’t know |
| 218 | HTN can cause Stroke, if left untreated. | 1. Yes 2. No 3. Don’t know |
| 219 | HTN can cause kidney failure, if left untreated. | 1. Yes 2. No 3. Don’t know |
| 220 | HTN can cause premature death, if left untreated. | 1. Yes 2. No 3. Don’t know |
| **E. Knowledge towards HTN treatment methods** | | |
| 221 | Herbal medications used to control HTN. | 1. Yes 2. No 3. Don’t know |
| 222 | Chemical drugs used to control HTN. | 1. Yes 2. No 3. Don’t know |
| 223 | Taking healthy diet used to control HTN. | 1. Yes 2. No 3. Don’t know |
| 225 | Smoking and alcohol cessation used to control HTN. | 1. Yes 2. No 3. Don’t know |
| 226 | Performing regular exercise used to control HTN. | 1. Yes 2. No 3. Don’t know |
| **Part III. Behavioral characteristics** | | |
| **A. Cigarette smoking** | | |
| 301 | Do you smoke cigarettes? | 1. Yes 2. No |
| **Note:** If your answer is **Yes** for question number 301 go to question number 302 and 303 | | |
| 302 | How frequently do you smoke? | 1. Daily 2. Once /wk. 3. 2 -3 days/wk. 4. 4-5 days/wk. 5. Other specify---------------- |
| 303 | How many cigarettes do you smoke at a time? | 1. 1 or less cigarette 2. 2-5 cigarettes 3. 6-10 cigarettes 4. 11 and more cigarettes |
| **B. Khat chewing** | | |
| 304 | Do you have a habit of Khat chewing? | 1. Yes 2. Nn |
| **Note:** If your answer is **Yes** for question number 304 go to question number 306 | | |
| 305 | How frequently do you chewing the khat? | 1. Daily 2. Most week days 3. Weekends only 4. On occasions |
| **C. Alcohol drinking** | | |
| 306 | Do you drink alcohol? | 1. Yes 2. No |
| **Note:** If your answer is **Yes** for question number 306 go to question number 307 and 308. | | |
| 307 | How often do you take alcoholic drinks? | 1. Daily 2. 5-6 days per week 3. 1-4 days per week 4. 1-3 days per week 5. Other specify--------------- |
| 308 | On average, how much do you usually drink alcohol? (one portion of alcohol is having at least 1 glass of wine, 1 bottle of beer, a 50g of ouzo) | 1. Less than one drink 2. One to three drinks 3. Four to six drinks 4. Seven or more drinks |
| **D. Dietary history** | | |
| 309 | Do you eat fruits? | 1. Yes 2. No |
| **Note:** If your answer is **Yes** for question number 309 go to question number 310 and 311 | | |
| 310 | In a typical week, on how many days do you eat fruit? | 1. Daily 2. 1- 4 days per week |
| 311 | How many servings of fruits do you eat on one of those days? (1 serving= one orange/ apple/banana/peach/mango/grapes etc.). | 1. 4 serving of fruits 2. 5 or more servings of fruits |
| 312 | How often you have eaten under stressful condition? | 1. 1. Daily 2. 2 .occasionally 3. 3. Some times |
| 313 | How often you have eaten while driving? | 1. Daily   1. .occasionally 2. Some times 3. Never |
| 314 | How often do you eat high calorie diet such as egg, raw meat and chicken? | 1. 1. Daily 2. 2 .occasionally 3. 3. Some times |
| 315 | Do you eat vegetables? | 1. 1. Yes 2. No |
| **Note:** If your answer is **Yes** for question number 315 go to question number 316 and 317 | | |
| 316 | In a typical week, on how many days do you eat vegetables? | 1. Daily 2. 1- 4 days per week |
| 317 | How many servings of vegetables do you eat on one of those days? (1 serving= three tablespoons of cooked vegetables) | 1. 1. 4 serving of vegetables 2. 2. 5 or more servings of vegetables |
| **E. Physical activity** | | |
| 318 | Do you perform regular physical exercise? | 1. Yes  2. No |
| **Note:** If your answer is **Yes** for question number 318 go to question number 319 through 321 | | |
| 319 | What type of exercise do you perform? | 1. Walking 2. Jogging 3. Cycling 4. Swimming |
| 320 | How often do you exercise? | 1. < 5 days per week 2. ≥ 5 days per week |
| 321 | For how many minutes do you exercise per session? | 1. <30 minutes 2. ≥30 minutes |
| 322 | For how long hours you drive per day? |  |
| 322 | Do you have rest breaks interruption while driving? | 1. A. Yes 2. B. No |
| 323 | On average how many times do you interrupt per journey? |  |
|  | If your answer is Yes to question 322 go to question 323 |  |
| 401 | Do you have family history of HTN? | 1. Yes 2. No 3. I don’t know |
| **Note:** If your answer is **Yes** for question number 401 go to question number 402 | | |
| 402 | Who of your family has HTN? | 1. Father 2. Mother 3. Grand father 4. Grand mother |
| 403 | Have you ever been told by a doctor that you have diabetes mellitus? | 1. Yes 2. No |
| 404 | Have you ever been told by a doctor that you have cardiovascular problem? | 1. Yes 2. No |

| Part V. Blood Pressure level and Body Mass Index | | |
| --- | --- | --- |
| A. Blood Pressure level | | |
| 501 | First blood pressure measurement | Systolic--------------mmHg |
|  |  | Diastolic------------ mmHg |
| 502 | Second blood pressure measurement | Systolic--------------mmHg |
|  |  | Diastolic-------------mmHg |
| 503 | Third blood pressure measurement | Systolic--------------mmHg |
|  |  | Diastolic-------------mmHg |
| 504 | Average blood pressure measurement | Systolic--------------mmHg |
|  |  | Diastolic-------------mmHg |
| A. Height and Weight | | |
| 505 | Weight | --------------------------kg |
| 506 | Height | --------------------------Meter |
| 507 | BMI level | -------------------------kg/m^2^ |

WE THANK YOU FOR THE FULL COOPRERATION!

SUPPERVISOR NAME------------------------- SIGNATURE-----------DATE----------------
